# Navigating Viral Gastroenteritis: Epidemiological Trends, Pathogen Analysis, and Histopathological Findings

**DOI:** 10.7759/cureus.61197

**Published:** 2024-05-27

**Authors:** Poonam C Sharma, Martin McCandless, Sumit P Sontakke, Neha Varshney, Robert T Brodell, Patrick B Kyle, William Daley

**Affiliations:** 1 Pathology, University of Mississippi Medical Center, Jackson, USA; 2 Neurosurgery, University of Kansas Medical Center, Kansas City, USA; 3 Medical Foundations, Ross University School of Medicine, Bridgetown, BRB; 4 Pathology and Dermatology, University of Mississippi Medical Center, Jackson, USA

**Keywords:** viral gastroenteritis, sapovirus, rotavirus, norovirus, astrovirus, adenovirus

## Abstract

Background: Gastroenteritis is a common cause of morbidity and mortality globally. Its cause encompasses a spectrum of agents, including viruses, bacteria, parasites, toxins, and drugs. Viruses account for a considerable portion of gastroenteritis cases across all age groups, typically presenting with symptoms like nausea, vomiting, diarrhea, dehydration, anorexia, and weight loss. While sporadic cases occur, viral gastroenteritis is more frequently observed in outbreaks within closely knit communities such as daycare facilities, nursing homes, and cruise ships. Therefore, it becomes necessary to determine when healthcare providers should consider this condition in their differential diagnosis and to develop the most effective strategy to confirm the diagnosis.

Methods: De-identified data of patients with gastroenteritis were collected over a five-year period utilizing the Patient Cohort Explorer, an electronic health record at the University of Mississippi Medical Center. Confirmatory laboratory tests employed the BioFire® FilmArray® multiplex polymerase chain reaction for gastrointestinal pathogens. Out of the 22 most common agents associated with gastroenteritis, only viral pathogens, specifically adenovirus, astrovirus, norovirus, rotavirus, and sapovirus, were included in the analysis. When available, histopathology was reviewed.

Results: Among the various causes of gastroenteritis, both infectious and non-infectious, our findings revealed that 25.46% of the cases were linked to viral pathogens. This included a significantly higher percentage of pediatric patients (72.73%) when compared to adults (27.07%), with a p-value of 0.015. Norovirus genogroups I and II emerged as the most frequently detected viruses across all age groups, with a significant prevalence among adults. No discernible gender-based differences were observed. The histopathological findings included inflammation, ulceration, erosion, architectural distortion, and the pathognomonic viral inclusion bodies associated with adenovirus.

Conclusion: Our comprehensive analysis of viral gastroenteritis cases highlights the substantial burden of this condition, particularly among pediatric patients. Norovirus emerges as a prevalent culprit which emphasizes the importance of vigilant surveillance and timely diagnosis, especially in settings where outbreaks are common.

## Introduction

Gastroenteritis remains a significant public health concern, posing an immense burden on healthcare systems globally. The World Health Organization estimates that diarrheal disease, often a hallmark of gastroenteritis, is the second leading cause of death in children under five years old, accounting for about 525,000 deaths annually [1]. Although the condition affects populations worldwide, it disproportionately affects geographic areas where access to clean water and sanitation facilities is limited [2]. The etiological factors of gastroenteritis are multifaceted and can be broadly categorized into infections and non-infectious agents. Infectious causes include bacteria (e.g., *Salmonella*, *Campylobacter*), parasites (e.g., *Giardia*, *Cryptosporidium*), and viruses (e.g., norovirus, rotavirus) [3]. Non-infectious causes range from drug-induced enteropathy to toxic ingestions [4]. Among these, viral agents are increasingly recognized for their substantial contribution to the overall morbidity and mortality related to gastroenteritis [5].

Globally, viruses are the predominant cause of acute gastroenteritis [6-8]. The majority of cases of acute viral gastroenteritis (VGE) are caused by rotaviruses, caliciviruses, enteric adenoviruses, and astroviruses. While most are spread through the fecal-oral route, transmission has also been demonstrated by fomites, vomitus, or airborne pathways [9]. Typical clinical manifestations of viral gastroenteritis include nausea, vomiting, diarrhea, dehydration, anorexia, and weight loss. These symptoms, although non-specific, often warrant medical evaluation and, in severe cases, hospitalization [10]. Moreover, outbreaks of viral gastroenteritis are commonly reported in closed or semi-closed communities, including daycare centers, nursing homes, and cruise ships [11]. These outbreak-prone settings necessitate vigilant surveillance and timely diagnosis, as the rapid spread of viral agents can lead to substantial public health crises [12].

Diagnostic methods for the VGE often involve molecular techniques such as polymerase chain reaction (PCR) or enzyme-linked immunosorbent assay (ELISA) for rapid and accurate identification [13]. For adenovirus, antiviral agents like cidofovir have been utilized in severe cases, although supportive therapy remains the mainstay [14]. Astrovirus, norovirus, and sapovirus typically require only symptomatic treatment, and no specific antiviral therapies exist [15,16]. For rotavirus, vaccines, such as Rotarix® and RotaTeq®, have demonstrated efficacy in reducing the severity of the disease [17].

Given the need for continued public health surveillance, the current study aims to provide a comprehensive analysis of patients with viral gastroenteritis, focusing on its epidemiology, age distribution, and predominant viruses involved. Specifically, the role of five commonly encountered viral pathogens, adenovirus, astrovirus, norovirus, rotavirus, and sapovirus, was examined in patients diagnosed with gastroenteritis over a five-year period. The objective was to identify patterns to aid clinicians in considering viral gastroenteritis in their differential diagnosis and optimizing their diagnostic approach.

## Materials and methods

Study design

In this retrospective observational study, the de-identified patient data were obtained from the University of Mississippi Medical Center's (UMMC) Patient Cohort Explorer (PCE), a Health Insurance Portability and Accountability Act (HIPAA) de-identified electronic data warehouse including more than 900,000 patients and 33 million patient encounters at the UMMC. 

Patient selection

Individual outpatients and inpatients receiving care at the UMMC from January 2018 to June 2023 were included. Additional encounters with these same patients were excluded.

Data collection

The patient data from the diagnosis group "Gastroenteritis" and lab components tested for adenovirus types F40/41, astrovirus, norovirus genogroups I/ II, rotavirus group A, and sapovirus genogroups I, II, IV, and V were obtained. These were detected using the BioFire® FilmArray® multiplex polymerase chain reaction (PCR) for gastrointestinal pathogens. This panel includes the targets for the 22 most common agents associated with gastroenteritis with a sensitivity of 98.5% and a specificity of 99.2% [18]. Histopathologic sections from the gastrointestinal tract stained with hematoxylin and eosin were reviewed when available.

Standard protocol approvals and patient consents

The data was obtained from the UMMC's PCE with removed HIPAA identifiers and dates adjusted by a random number of days with seasonality taken into consideration. Institutional review board approval was not obtained in accordance with 45 CFR 46 federal regulations.

Statistics

The data were plotted using the Microsoft® Excel 2019 Office program, and the statistical analysis was done using GraphPad Prism® (Version 10.0, GraphPad Software, www.graphpad.com, accessed on 17 September 2023). The data were evaluated using one-way ANOVA for multiple comparisons by Tukey test as well as student t-tests between two groups in GraphPad Prism 10.0. The differences between various groups were considered significant when a p-value was less than 0.05. The standard deviation and the 95% confidence interval were also calculated using GraphPad Prism.

## Results

Seven hundred and eleven gastroenteritis patients with both infectious and non-infectious etiologies were included in this study. Among the various causes of gastroenteritis, viral pathogens accounted for 25.5% of the cases (Table 1). The pediatric population (0-17 years of age) was infected more commonly (72.7%) when compared to adult cases (27.1%) (p-value of 0.015) (Table 2). No difference between male and female populations was observed (Table 3).

**Table 1:** Detection of viral isolates from the gastroenteritis patients

Number of stool samples positive for virus panel	Total number of stool samples from gastroenteritis cases processed	Percentage of stool samples positive for virus panel
181	711	25.46

**Table 2:** Comparison between pediatric and adult viral gastroenteritis population CI: confidence interval; SD: standard deviation

Year	2023	2022	2021	2020	2019	Mean (%)	Lower 95% CI	Upper 95% CI	SD (%)	P-value
Children (%)	44	75.5	78.79	57.14	100	71.09	44.43	97.74	21.47	0.015
Adult (%)	56	24.5	21.21	42.86	0	28.91	2.26	55.57		

**Table 3:** Gender-based differences among the viral gastroenteritis patients CI: confidence interval; SD: standard deviation

Year	2023	2022	2021	2020	2019	Mean (%)	Lower 95% CI	Upper 95% CI	SD (%)	P-value
Females (%)	52	45.1	57.58	42.86	57.14	50.94	42.54	59.34	3.025	0.6733
Males (%)	48	54.9	42.42	57.14	42.86	49.06	40.66	57.46		

**Figure 1:**
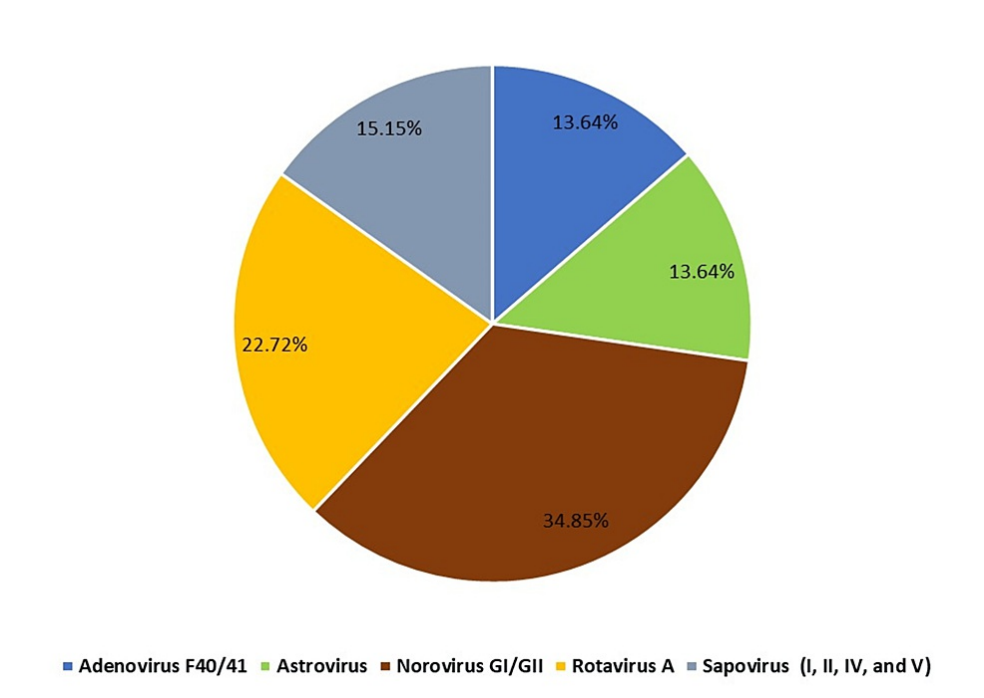
Distribution of viral pathogens detected from included pediatric individuals who had at least one virus detected

**Figure 2:**
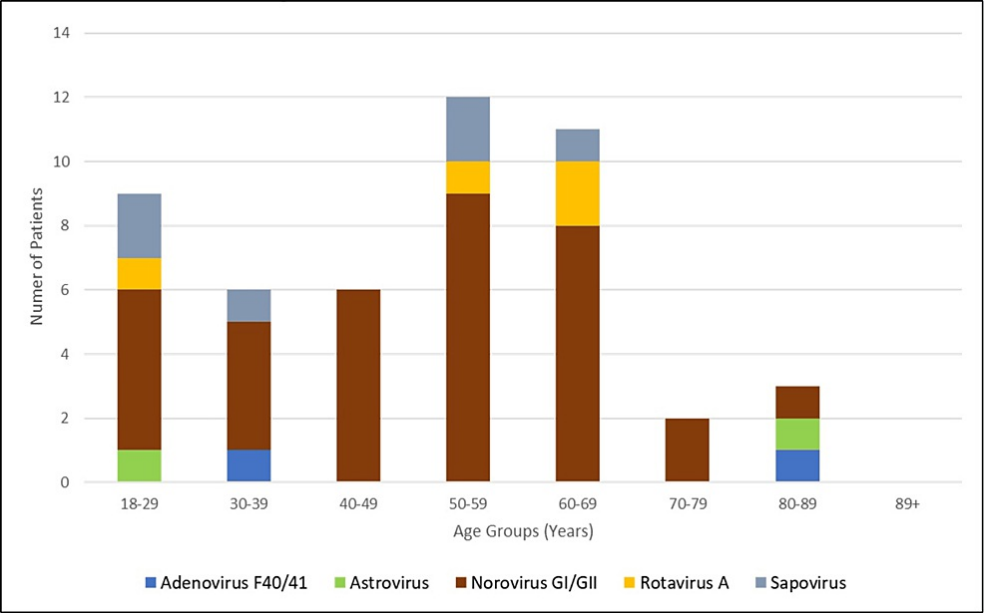
Age distribution of viral pathogens detected from included adult individuals who had at least one virus detected

Viral pathogens were assessed in both adult and pediatric age groups. Norovirus genogroups I and II were mostly frequently detected in both of these groups (Figure 1), but the noroviral cases were only significantly high in adults (adjusted p-value <0.001) (Table 4). To determine if the characteristics of viral infection vary by age, the adult population was divided into age groups. Individuals aged 50-69 years were most commonly infected among the adult VGE cases (Figure 2).

**Table 4:** Pattern of viral isolates in adult population with gastroenteritis SD: standard deviation

Viral pathogen in adults	Mean (%)	SD (%)	Comparison of norovirus cases with each virus isolated (adjusted p-value)
Adenovirus F40/41	7.143	13.11	<0.001
Astrovirus	6.349	12.60	<0.001
Norovirus GI/GII	71.90	23.71	-
Rotavirus	5.374	7.32	<0.001
Sapovirus (I, II, IV, and V)	9.237	9.44	<0.001

**Figure 3:**
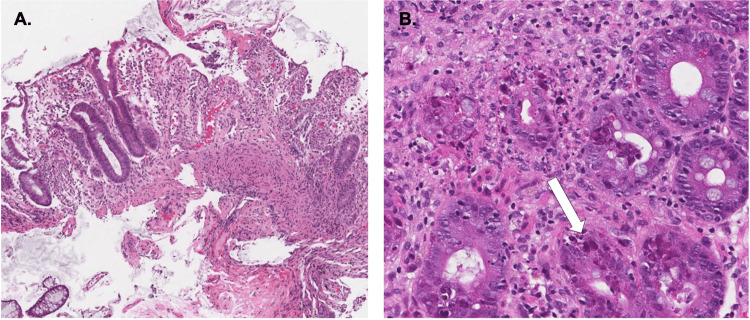
Histopathology section of the intestine Hematoxylin and eosin-stained slides of the intestine show (A) extensive mucosal ulceration and (B) numerous apoptotic crypt epithelial cells and eosinophilic intranuclear inclusions (as indicated by white arrow) which are typical features of adenoviral infection.

The histopathological examination revealed a range of alterations including acute inflammatory cellular infiltrates, mucosal ulceration, surface erosion, architectural distortion, and the characteristic viral inclusion bodies. These inclusion bodies, with their distinctive morphological features, serve as hallmarks of adenovirus infection, further corroborating the diagnosis (Figure 3). 

## Discussion

Precise information regarding the frequency and characteristics of sporadic gastroenteritis cases is limited. This is primarily due to the mild nature of these illnesses, which often do not necessitate medical attention. Individuals might not undergo diagnostic testing even if they seek medical care. In addition, depending on the specific pathogen involved, reporting through public health surveillance systems is not required. This study, based on a five-year analysis of de-identified data from the UMMC, provides valuable insights into the epidemiology of viral gastroenteritis and the distribution of specific viral pathogens. The overall occurrence of viral gastroenteritis in our study was 25.5%, underscoring the substantial burden of this condition in this population. This is consistent with the research conducted by Schmidt et al. [19]. They observed that 22.4% of the stool samples from individuals reporting acute gastroenteritis yielded positive results for at least one viral pathogen.

Clinicians encounter acute VGE in three distinct scenarios. The first involves sporadic gastroenteritis in children, primarily attributed to rotaviruses [20]. The second scenario is epidemic gastroenteritis, occurring in semi-closed communities, such as families, institutions, cruises, and vacation spots, or resulting from classic food-borne or water-borne pathogens, with caliciviruses being the primary culprits [21]. The third scenario involves sporadic acute gastroenteritis in adults, with caliciviruses, adenoviruses, rotaviruses, or astroviruses being the most likely causative agents [22].

Notably, pediatric patients in this study accounted for a considerable majority of VGE (72.7%), emphasizing the vulnerability of this demographic group to viral pathogens associated with gastroenteritis. This finding highlights the heightened susceptibility of children to gastrointestinal infections [23,24], likely due to factors such as immature immune system and increased social interactions in environments like daycare facilities. Norovirus, belonging to genogroups I and II, emerged as the most frequently detected virus across all age groups, with a significant prevalence among adults. This observation is consistent with the well-documented role of norovirus as a major cause of both sporadic cases and outbreaks of gastroenteritis in various settings [25]. The highly contagious nature of norovirus, coupled with its resistance to environmental factors, contributes to its sustained prevalence in communities and healthcare institutions [26]. 

Globally, rotavirus stands as the primary pathogen responsible for severe diarrhea in children [27]. In this research, the number of rotavirus cases was surpassed by norovirus infections in children. This could be attributed to the fact that Mississippi has the highest pediatric vaccination rate in the United States [28,29]. Therefore, the widespread administration of the rotavirus vaccine might contribute to a decrease in the rotaviral infection rate. Also, the gender distribution of viral gastroenteritis cases did not reveal discernible differences, suggesting that susceptibility to viral pathogens causing gastroenteritis is not strongly influenced by the patient's sex. This finding aligns with previous research indicating that viral gastroenteritis affects both males and females equally [19]. This study was conducted retrospectively using data from a single academic medical center. This might limit the generalizability of the findings to other populations or regions.

This study utilized the BioFire® FilmArray® multiplex PCR for gastrointestinal pathogens, offering a comprehensive approach to identify a spectrum of viral agents. In contrast to conventional diagnostic approaches, syndromic testing by multiplex PCR can decrease the average number of laboratory tests, lower the number of send-out tests, and cut down the average time to result [30]. Thus, it provides a number of clinical benefits including decreased use of antibiotics, facilitation of targeted therapy, and a decreased need for downstream procedures such as abdominal imaging and endoscopies [31,32].

In this investigation, significant emphasis was placed on the histopathological findings. They can play a crucial role in discerning the presence of infection. This is particularly important given the backdrop of numerous device recalls associated with the BioFire® Diagnostics gastrointestinal panel, attributed to a high incidence of false-negative or false-positive outcomes [33,34]. False-negative results may precipitate inappropriate antibiotic administration, lack of treatment, treatment delay, and the potential provision of false reassurance to individuals harboring a true infection. Additionally, they may impede the timely identification of outbreaks that contribute to further disease transmission. Conversely, false-positive outcomes can lead to unnecessary treatment and diminished prospects of identifying the true etiology of the patient's ailment. The risk posed by such outcomes, however, can be mitigated by comprehensively evaluating other clinical and diagnostic indicators, including the patient's clinical history, travel history, clinical evidence of infection, and disease severity. There is a critical need to correlate these findings with histopathological evidence to ensure accurate diagnosis and appropriate management.

Before the advent of rapid nucleic acid testing, diagnostic testing and differentiation were primarily conducted for epidemiological purposes. Nonetheless, individuals exhibiting alarm symptoms or signs such as severe dehydration, electrolyte imbalance, renal dysfunction, severe abdominal pain, and symptoms lasting more than one week and old-age, pregnant, or immunocompromised patients should undergo stool testing [23]. The use of advanced diagnostic tools enhances the accuracy of identifying causative agents, aiding in the timely implementation of appropriate management and infection control measures. Timely diagnosis of viral gastroenteritis is critical, especially in settings prone to outbreaks. The high prevalence of norovirus underscores the importance of vigilant surveillance, rapid diagnosis, and implementation of preventive measures to curtail the spread of the virus in environments such as daycare facilities, nursing homes, and other closely knit communities.

## Conclusions

The results of this study heighten the understanding of the clinical landscape of viral gastroenteritis at an academic medical center. It accentuates the high infection among pediatric patients and underscores norovirus as a commonly encountered viral pathogen, particularly in adults. These findings strongly advocate for continued research and policy intervention focused on viral gastroenteritis, aimed at enhancing diagnostic accuracy, patient management, and public health responses. Further studies should delve deeper into this area to assess the effectiveness of current and future preventive measures, such as vaccines or improved sanitation protocols, to mitigate the impact of viral gastroenteritis.
